# Targeting Bladder Cancer with Inactivated Uropathogenic *E. coli*: A Novel Alternative to BCG Immunotherapy

**DOI:** 10.3390/cells15030229

**Published:** 2026-01-26

**Authors:** Vladimir Yutkin, Naseem Maalouf, Chamutal Gur, Avraham Zini, Gilad Bachrach, Ofer Mandelboim

**Affiliations:** 1 Department of Urology, Hadassah Hebrew University Medical School, Jerusalem 91120, Israel; 2 The Institute of Dental Sciences, The Hebrew University-Hadassah School of Dental Medicine, Jerusalem 91120, Israel; 3 Department of Oral and Maxillofacial Surgery, Tzafon Medical Center, Tveria 1520801, Israel; 4 The Concern Foundation Laboratories, The Lautenberg Center for Immunology and Cancer Research, Hebrew University Hadassah Medical School (IMRIC), Jerusalem 91120, Israel; 5 The Rheumatology Research Center, Hadassah-Hebrew University, Jerusalem 91120, Israel; 6 Department of Community Dentistry, Hadassah Medical Center, Faculty of Dental Medicine, Hebrew University of Jerusalem, Jerusalem 91120, Israel

**Keywords:** bladder cancer, BCG

## Abstract

More than 90% of bladder cancers are classified as urothelial carcinomas (UC), with approximately 75% of these cases presenting as non-muscle-invasive bladder cancer (NMIBC). Bacillus Calmette–Guérin (BCG) is the current standard immunotherapy for NMIBC, yet it suffers from limited efficacy, frequent tumor recurrence, and substantial toxicity. These limitations underscore the need for safer, more effective, and accessible alternatives. We investigated whether uropathogenic *Escherichia coli* (UPEC), a natural inducer of immune responses in the bladder, could serve as a novel intravesical immunotherapeutic agent. Using orthotopic bladder cancer models in both mice (MB49-luc) and rats (AY-27), we evaluated the efficacy, specificity, immune dependence, and safety of formaldehyde-inactivated UPEC strains, including mutants with altered type 1 fimbriae expression. Intravesical administration of inactivated UPEC significantly reduced tumor burden and prolonged survival, outperforming BCG in murine models and demonstrating equivalent efficacy with markedly reduced toxicity in rats. The antitumor effect was T cell-dependent and partially mediated by type I fimbriae, which facilitated tumor-specific adhesion. Notably, systemic (subcutaneous) administration of UPEC abrogated efficacy and increased mortality, emphasizing the necessity of localized bladder delivery. In conclusion, we identify inactivated UPEC as a potent, tumor-targeting, and T cell-dependent immunotherapeutic agent with a superior safety profile compared to BCG. This approach might represent a promising and practical alternative for bladder cancer treatment.

## 1. Introduction

The use of bacteria to enhance antitumor immunity dates back to 1891, when William Coley successfully treated sarcomas with extracts derived from pyogenic bacteria, an approach that laid the foundation for bacterial cancer immunotherapy [1]. Coley also observed an inverse correlation between tuberculosis (TB) infection and cancer incidence, a finding that sparked interest in harnessing live bacteria for cancer treatment [2].

Building on this concept, Holmgren reported in 1935 that Mycobacterium Bovis Bacillus Calmette–Guérin (BCG), a live attenuated TB vaccine, could prevent bladder and other tumor malignancies [3]. This led to the development of intravesical BCG as a standard adjuvant therapy for non-muscle-invasive bladder cancer (NMIBC), following tumor resection. Since the mid-1980s, BCG has supplanted cystectomy as the treatment of choice for high-risk NMIBC [4].

Bladder cancer is the fourth most common cancer among men in Europe and the U.S., with a male-to-female incidence ratio of approximately 3:1 [5,6]. Interestingly, the prevalence of urinary tract infections caused by uropathogenic *Escherichia coli* (UPEC) displays the opposite pattern, affecting women more frequently. The reasons for these gender-specific differences remain unclear [7]. Over 90% of bladder tumors are urothelial carcinomas (UC), and about 75% present as NMIBC [8]. While transurethral resection remains the first-line intervention, recurrence rates exceed 70%, and 10% to 20% of cases progress to muscle-invasive disease [8].

According to meta-analysis, BCG improved recurrence-free rate from 26% to 47% and progression-free rate from 15% to 20% [9]. However, its efficacy is limited, with many high-grade tumors eventually recurring or progressing [8]. Additionally, up to 60% of patients experience significant adverse effects, including potentially life-threatening infections, leading to treatment discontinuation [8]. These limitations underscore the urgent need for safer and more effective immunotherapeutic alternatives.

Despite decades of clinical use, the mechanism underlying BCG’s antitumor activity remains incompletely understood [10]. Both innate and adaptive immune responses, including those mediated by natural killer (NK) and T cells, are essential for BCG’s efficacy [11,12,13]. Yet, it is unclear whether this immune activation is specific to BCG or whether alternative bacteria might similarly stimulate protective responses in the bladder [14].

In previous work, we demonstrated that the healthy bladder is largely devoid of immune cells, but that infection with UPEC triggers the secretion of SDF1 and the consequent recruitment of NK and T cells [15,16,17]. Notably, UPEC subverts this immune infiltration by producing hemolysinA, a toxin that selectively kills NK cells [15]. Using a transposon mutagenesis screen, we identified a hemolysinA-deficient UPEC mutant (C93) that fails to eliminate NK cells and leads to greater immune cell accumulation in the bladder compared to wild-type UPEC CFT073 strain [15]. Given the immune dependence of BCG efficacy and the gender-inverted patterns of UPEC infection and UC incidence, we hypothesized that engineered or attenuated UPEC strains may serve as novel, immune-stimulating agents for bladder cancer treatment.

## 2. Results

### 2.1. The Hemolysin-Deficient C93 Mutant Is More Efficient than BCG in Preventing TCC Development

BCG therapy is known to rely on both T and NK cell activity for its antitumor efficacy [12,13]. We have previously shown that the hemolysinA-deficient UPEC mutant C93 recruits NK and T cells to the bladder while lacking the ability to eliminate them, in contrast to the wild-type strain that expresses hemolysinA [15]. Based on these findings, we hypothesized that C93 might serve as a superior immunotherapeutic agent compared to BCG for the treatment of bladder cancer.

To test this, we employed the MB49-luc (MB49 cells stably expressing luciferase) orthotopic bladder cancer model in female C57BL/6 mice. MB49-luc bladder carcinoma cells (3 × 10^4^) were implanted orthotopically into female C57BL/6 mice following electrocautery-assisted bladder wall preparation, as described in Section 4. Three days after tumor implantation, mice received the first intravesical instillation of inactivated C93 UPEC, BCG, or PBS (control). Treatments were administered once weekly via catheterization. Mice were monitored for survival and clinical signs of disease up to day 63 post-implantation, and the presence of tumors was confirmed post mortem.

**Figure 1:**
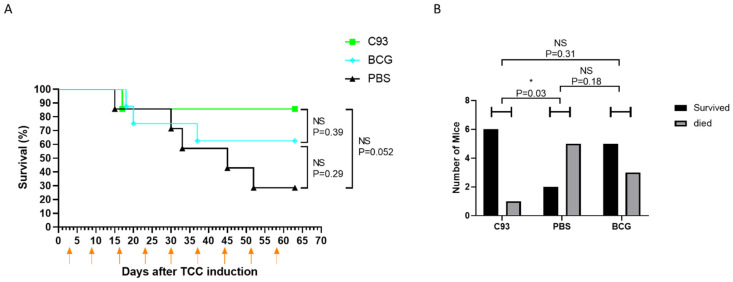
The HemolysinA mutant C93 is effective against TCC. Bladders of 7–8-week-old female C57BL/6 mice were orthotopically implanted with MB49 cancer cells stably transfected with luciferase (MB49-luc, 3 × 10^4^ cells/mouse). Tumor growth was monitored by CCD camera, and mice were grouped according to tumor size (7–8 per group). Following tumor inoculation, mice were treated with either 10^8^ CFU C93 UPEC mutant, 1–4 × 10^8^ CFU of BCG, or PBS (negative control). The first treatment was administered 3 days after tumor inoculation and repeated weekly (as indicated by orange arrows in (**A**)). (**A**) Kaplan–Meier curve with log-rank testing showing mouse survival from tumor implantation until day 63. (**B**) Summary of the total number of treated mice in each group (X-axis), subdivided into survivors (black columns) and non-survivors (gray columns). Statistical comparisons were performed using the chi-square test, which incorporated both survival and death subgroups. NS = not significant, * *p* < 0.05.

Strikingly, C93 treatment significantly outperformed control (PBS) in reducing mortality and preventing tumor development (Figure 1).

### 2.2. Formaldehyde-Inactivated UPEC Retains Antitumor Activity in the MB49-Luc Bladder Cancer Model

**Figure 2:**
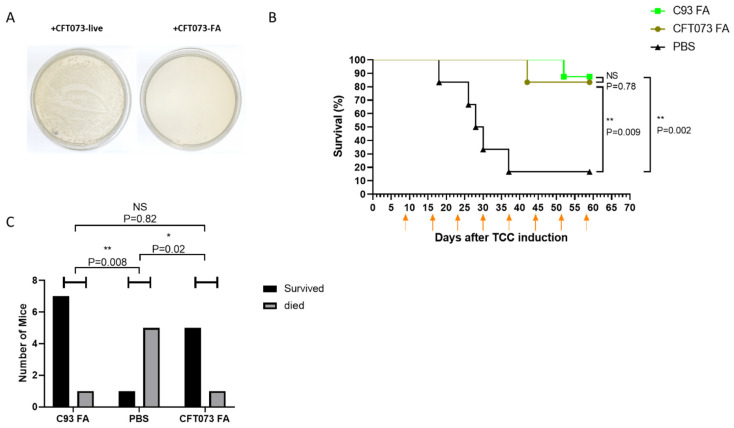
Formaldehyde (FA)-inactivated UPEC is effective against TCC. (**A**) UPEC C93 and wild-type CFT073 strains were either left untreated or treated with 4% formaldehyde. To confirm bacterial inactivation, 100 µL of OD = 1 suspensions were plated on LB agar and incubated overnight. Bacterial growth was assessed after incubation. (**B**) Female C57BL/6 mice (6–8 per group) were orthotopically implanted with MB49-luc bladder carcinoma cells. Ten days after tumor inoculation, mice received intravesical treatments with PBS (negative control), FA-inactivated wild-type UPEC (CFT073 FA), or FA-inactivated hemolysin-deficient C93 (C93 FA) mutant (10^8^ CFU equivalent/dose). Treatments were repeated weekly (orange arrows in (**B**)). Survival curves show survival from tumor implantation until day 60. Data is presented as Kaplan–Meier curve with log-rank testing. (**C**) Summary of treated mice in each group (X-axis), subdivided into survivors (black columns) and non-survivors (gray columns). Statistical analysis was performed using the chi-square test, incorporating both survival and death subgroups. NS = not significant, * *p* < 0.05, ** *p* < 0.01.

We next assessed whether non-viable (formaldehyde-inactivated) UPEC could elicit antitumor activity against bladder cancer. Broth grown wild-type UPEC (CFT073) and the hemolysinA-deficient mutant (C93) were inactivated using 4% formaldehyde, as described in Section 4. To confirm inactivation, 100 µL of bacterial suspensions at OD600nm = 1 were plated on LB agar plates at 1:1, 1:10, and 1:100 dilutions, and incubated overnight at 37 °C. The suspensions were also cultured in LB broth under the same conditions. No bacterial growth was observed on plates or in liquid media, confirming inactivation (Figure 2A).

To evaluate the therapeutic potential of the inactivated strains, C57BL/6 mice were orthotopically inoculated with MB49-luc bladder carcinoma cells as described above. Tumor burden was assessed via IVIS on day 7, with treatments initiated at day 10 post-tumor inoculation. Mice received weekly instillations of formaldehyde-inactivated WT UPEC (CFT073), inactivated C93, or PBS control. Animals were followed for survival and tumor progression for up to 60 days post-implantation. As shown in Figure 2B,C, both inactivated strains achieved potent therapeutic effects, curing approximately 80–90% of the tumor-bearing mice. No significant difference was observed between the wild-type and mutant-treated strains. Based on these results, the subsequent experiments utilized formaldehyde-inactivated WT CFT073 as the therapeutic agent.

### 2.3. Inactivated UPEC Exhibits Specific Antitumor Activity Partially Mediated by Type I Fimbriae

**Figure 3:**
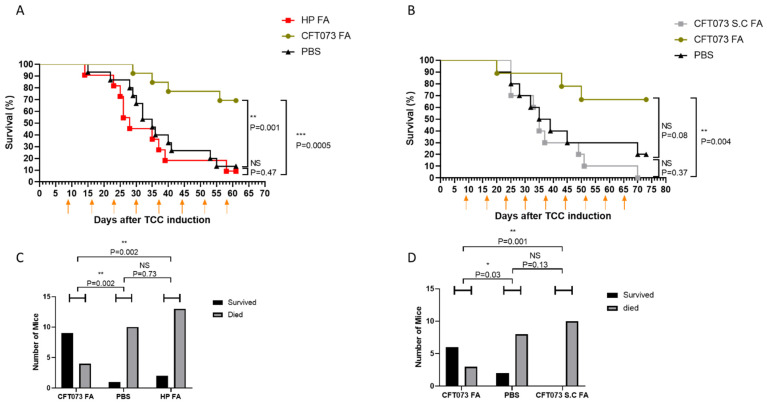
UPEC treatment is organ specific and requires intravesical administration. (**A**,**C**) C57BL/6 female mice (11–15 mice per group), were inoculated with MB49-luc bladder carcinoma cells. Following cancer inoculation, mice were treated intravesically with PBS (negative control), with 10^8^ FA-treated wild-type UPEC (CFT073 FA) bacteria or with FA-treated *Helicobacter pylori* (HP FA). (**B**,**D**) C57BL/6 female mice (8–11 mice per group) were implanted with MB49-luc bladder carcinoma cells. Mice were treated with 10^8^ FA-treated UPEC bacteria intravesically (CFT073 FA), or subcutaneously (CFT073 S.C. FA); or with PBS (negative control). The treatment days are marked by orange arrows in (**A**,**B**). Data in (**A**,**B**) is presented as Kaplan–Meier curve with log-rank testing. (**C**,**D**) Summary of treated mice (X-axis) in each group ((**A**) and (**B**), respectively), subdivided into survivors (black columns) and non-survivors (gray columns). Statistical analysis was performed using the chi-square test, incorporating both survival and death subgroups. NS = not significant, * *p* < 0.05, ** *p* < 0.01, *** *p* < 0.001.

To determine whether the observed antitumor effect of UPEC was specific or could be elicited by any bacterial species, we compared the effects of intravesical instillation of formaldehyde-inactivated UPEC (CFT073) and *Helicobacter pylori* (HP) in MB49-luc tumor-bearing mice. Female C57BL/6 mice were implanted orthotopically with MB49-luc tumor cells. Intravesical treatments began after tumor establishment and were administered weekly as follows: formaldehyde-inactivated WT UPEC, formaldehyde-inactivated *Helicobacter pylori*, or PBS. In the route-of-administration arm, mice received either intravesical or subcutaneous UPEC. Treatment timelines matched those illustrated by the arrows in Figure 3. Animals were monitored throughout for survival outcomes. While inactivated UPEC rescued approximately 80% of the treated animals, HP treatment had no effect on tumor progression (Figure 3A), suggesting that the antitumor activity of UPEC is specific and not a generalized response to bacterial exposure.

We next tested whether the route of administration influences therapeutic efficacy. Tumor-bearing mice were injected either subcutaneously (s.c.) or into the bladder with inactivated UPEC. Surprisingly, subcutaneous administration not only failed to inhibit tumor growth, but actually resulted in accelerated progression (Figure 3B). These findings indicate that the UPEC-induced antitumor effect requires local intravesical delivery to the tumor site.

Given the potent and consistent therapeutic effect of UPEC (70–90% survival, see Figure 1, Figure 2 and Figure 3), we sought to investigate the role of type I fimbriae, that possesses the FimH adhesin, which mediates UPECs’ binding to bladder epithelium and to tumor tissue in both murine models and humans [14]. Type I fimbriae expression is regulated by a phase-variable promoter that can exist in either an “on” or “off” orientation [18]. To directly assess the contribution of type I fimbriae to antitumor efficacy, we utilized two genetically UPEC mutants: FIM ON, with the fim promoter locked in the active orientation, and FIM OFF, with the promoter locked in the inactive orientation.

**Figure 4:**
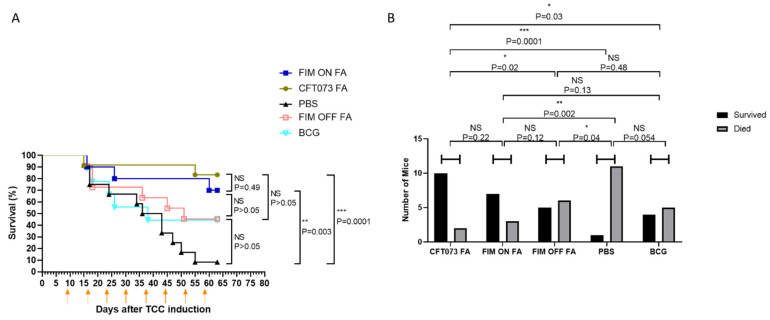
UPEC Treatment is partially FimH dependent. (**A**) C57BL/6 female mice (9–12 per group) were inoculated with MB49-luc bladder carcinoma cells. After cancer implantation, mice were treated intravesically with PBS (negative control), BCG, or with FA-treated wild-type UPEC (CFT073 FA), Fim ON (FIM ON FA) or Fim OFF (FIM OFF FA). Treatments started on day 9 post-cancer implantations and were repeated weekly (orange arrows). Data is presented as Kaplan–Meier curve with log-rank testing. (**B**) Outcomes for each treatment group (X-axis) are shown as survivors (black columns) and non-survivors (gray columns). Statistical comparisons were performed using the chi-square test, accounting for both survival and death subgroups. NS = not significant, * *p* < 0.05, ** *p* < 0.01, *** *p* < 0.001.

MB49-luc tumor cells were orthotopically inoculated into female C57BL/6 mice. Tumors were allowed to establish for 9 days before initiation of intravesical therapy. Mice were treated intravesically with formaldehyde-inactivated FIM ON or FIM OFF bacteria. As shown in Figure 4, FIM ON-treated mice exhibited survival rates comparable to those treated with wild-type CFT073, consistent with broth growth conditions favors expression of type I fimbriae in CFT073 [19,20,21]. In contrast, FIM OFF treatment was markedly less effective but still achieved a degree of tumor control comparable to that seen with live-attenuated BCG. The BCG group demonstrated high fluctuation in weight, indicating adverse side-effects (Supplementary Figure S1).

These results demonstrate that UPEC’s antitumor effect is both route-specific and partially dependent on its ability to express type I fimbriae, highlighting the importance of FimH-mediated bladder tumor targeting.

### 2.4. UPEC-Mediated Tumor Suppression Is T Cells Dependent

**Figure 5:**
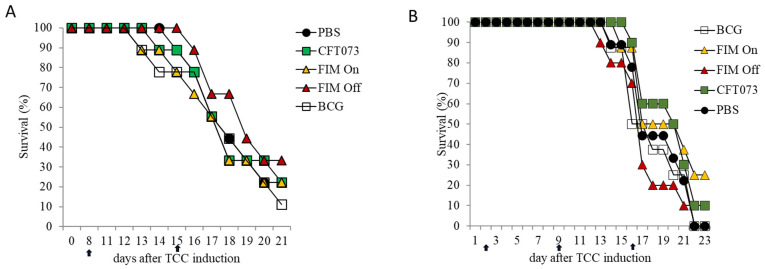
UPEC treatment is immune cell-dependent. Bladders of female Scid-Beige mice (8–9 mice per group) were inoculated with MB49-luc cells. Intravesical treatment with BCG (OncoTice), WT CFT073, Fim ON, Fim OFF, and PBS (negative control) started 8 (**A**), or 3 (**B**) days following tumor inoculation, and administered once a week (arrows).

To evaluate whether the antitumor activity of UPEC is immune cell-dependent, we employed SCID-Beige mice, which lack functional B, T, and NK cells. Female SCID-Beige mice received orthotopic MB49-luc tumor implantations. Tumors were allowed to establish either for 3 days (early treatment arm) or 8 days (late-treatment arm) before initiating weekly intravesical instillations of inactivated UPEC (FIM ON/OFF), BCG, or PBS. Mice were monitored daily post-inoculation, with survival followed until end-point criteria were reached (approximately day 21). MB49-luc bladder tumors grew aggressively in these immunodeficient mice, with nearly all animals succumbing by day 21 post-inoculation (Figure 5A). This rapid disease progression underscores the critical role of the immune system in controlling TCC growth. Importantly, neither UPEC (FIM ON) nor BCG treatment—administered either early (Figure 5B, day 3) or late (Figure 5A, day 8)—conferred any survival benefit in these mice, indicating that both therapies require host immune cells to exert their effects.

**Figure 6:**
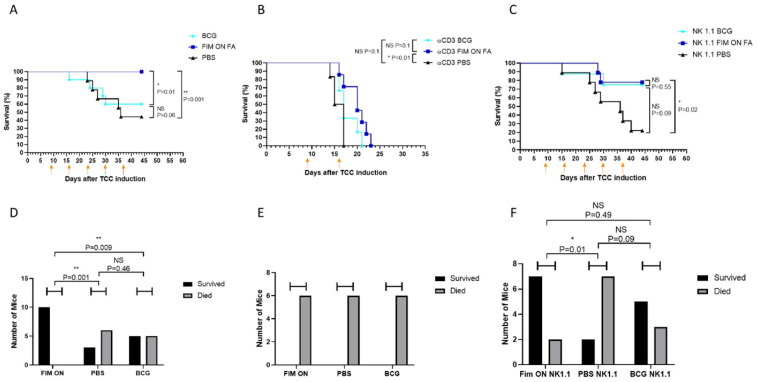
UPEC treatment is immune cell-dependent. (**A**–**C**) C57BL/6 female mice (7–10 mice per group), untreated (**A**), treated with anti-CD3 (**B**) or with anti-NK1.1 (**C**) were inoculated with MB49-luc cells. Nine days following cancer inoculation, mice were treated with BCG (BCG), FA-treated Fim ON (FIM ON FA), or with PBS (negative control). Treatments were repeated weekly (orange arrows). Data is presented as Kaplan–Meier curve with log-rank testing. (**D**–**F**) Summary of treated mice (X-axis) in each group ((**A**–**C**), respectively), subdivided into survivors (black columns) and non-survivors (gray columns). Statistical analysis was performed using the chi-square test, accounting for both survival and death subgroups. NS = not significant, * *p* < 0.05, ** *p* < 0.01.

To dissect the contribution of specific immune cell subsets, we next performed antibody-mediated depletion of T or NK cells in immunocompetent mice. Female C57BL/6 mice were implanted orthotopically with MB49-luc cells. Depleting antibodies (anti-CD3 or anti-NK1.1) were administered starting one day before treatment and then every three days thereafter. Nine days after tumor implantation, mice were treated intravesically once weekly with BCG, inactivated FIMON UPEC, or PBS. Survival and disease progression were monitored throughout the study period. Efficient depletion was confirmed by flow cytometry. In the absence of immune cell depletion, FIM ON-treated mice exhibited robust protection, with nearly 100% tumor-free survival, compared to 50–60% survival in BCG-treated mice (Figure 6A). Strikingly, depletion of T cells completely abrogated the therapeutic effect of both FIM ON and BCG, resulting in rapid tumor progression and death by day 21 (Figure 6B). In contrast, NK cell depletion had minimal impact on tumor control (Figure 6C), suggesting that NK cells are not essential for the efficacy of either treatment.

Together, these findings indicate that the antitumor activity of UPEC, like that of BCG, is critically dependent on T cells, while NK cells play a lesser or redundant role in this setting.

### 2.5. Inactivated UPEC Demonstrates Equivalent Efficacy to BCG and a Superior Safety Profile in the AY-27 Rat Bladder Cancer Model

**Figure 7:**
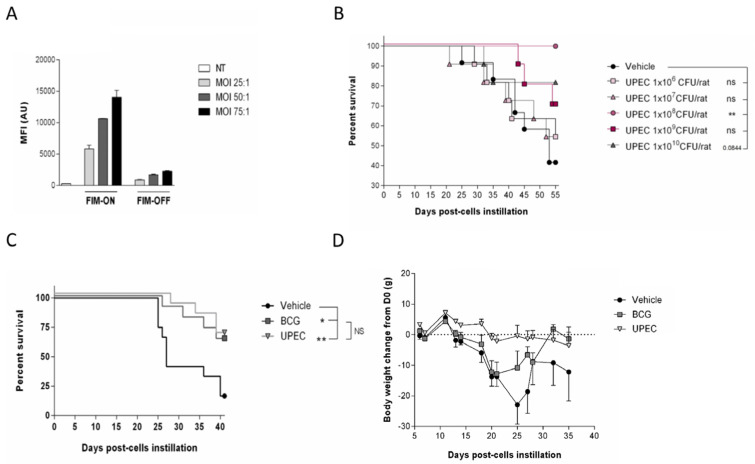
Inactivated UPEC is as efficient and more tolerable than BCG in the AY-27 rat bladder cancer model. (**A**) UPEC binding to AY-27 cells using FIM ON and FIM OFF strains. (**B**) Rats (9–12 per group) were inoculated intravesically with AY-27 bladder carcinoma cells. Six days after inoculation, rats were randomized into treatment groups and treated from day 7 twice weekly intravesically with vehicle (PBS, negative control) or with 1 × 10^6^–1 × 10^10^ formaldehyde-killed of UPEC Fim On. Data is presented as Kaplan–Meier survival curve * *p* < 0.05 and ** *p* < 0.01 vs. Vehicle, and NS *p* > 0.05 vs. BCG, Log-rank (Mantel–Cox) test. (**C**) Survival of rats post-AY-27 implantation, treated with control, 1.1–4.4 × 10^7^ CFU BCG and 10^8^ CFU equivalent UPEC. Data is presented as Kaplan–Meier survival curve * *p* < 0.05 and ** *p* < 0.01 vs. Vehicle, and NS *p* > 0.05 vs. BCG, Log-rank (Mantel–Cox) test. (**D**) Body weight of animals during the experiment presented in (**C**).

To further validate the therapeutic potential of inactivated UPEC, we tested its efficacy and safety in the immunocompetent rat AY-27 orthotopic bladder cancer model. Prior to in vivo evaluation, we assessed bacterial binding to AY-27 cells using the Fim ON and Fim Off UPEC strains. As shown in Figure 7A, Fim-ON UPEC exhibited higher binding to AY-27 cells across multiple MOIs (25:1, 50:1, and 75:1), confirming that type I fimbriae enhance UPEC adhesion to the rat bladder carcinoma cells. We next tested the therapeutic effect of inactivated UPEC using escalating doses of inactivated UPEC (10^6^–10^10^ CFU equivalent/rat) compared with vehicle control in rats bearing orthotopic bladder tumors. Treatments were administered intravesically twice weekly from day 7 to day 39 post-tumor cell instillation. A bell-shape effect was observed, low doses of UPEC (10^6^–10^7^ CFU equivalent/rat) did not significantly improve survival relative to vehicle. In contrast, higher doses (10^8^) resulted in a marked survival advantage, reaching statistical significance and showing a strong trend for 10^9^–10^10^ CFU equivalent (Figure 7B). These data demonstrate a clear dose-dependent therapeutic effect, with optimal activity observed at ≥10^8^ CFU/rat.

Next, rats were intravesically implanted with AY-27 tumor cells and treated twice weekly with either intravesical PBS vehicle, BCG (1–4 × 10^7^ CFU), or inactivated UPEC (1 × 10^8^ CFU equivalent). Survival analysis demonstrated that UPEC treatment significantly extended survival compared to vehicle control and was equivalent to BCG in its therapeutic efficacy (Figure 7C).

To assess treatment safety, we monitored body weight over time as an indicator of systemic toxicity. While BCG-treated rats experienced marked weight loss beginning around day 15 and persisting through day 30, UPEC-treated animals maintained stable body weight throughout the study period, closely resembling the untreated control group (Figure 7D).

These data confirm that inactivated UPEC is as effective as BCG in treating bladder cancer in the rat model, while offering a markedly improved safety profile with minimal systemic side effects.

**Figure 8:**
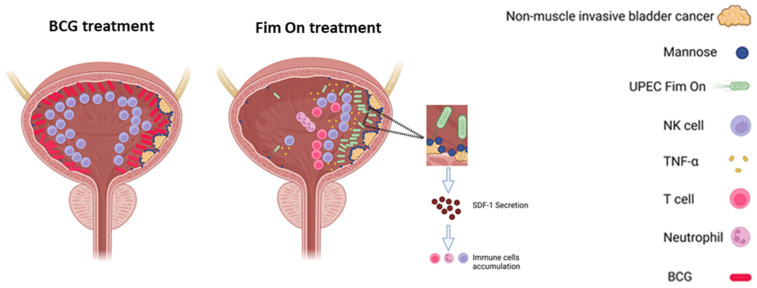
Proposed mechanism of bladder tumor eradication by intravesical instillation of formaldehyde-inactivated Fim ON UPEC. Schematic representation of the proposed immunological mechanism underlying bladder tumor control following intravesical administration of formaldehyde-inactivated, type 1 fimbria-expressing uropathogenic *Escherichia coli* (UPEC, Fim ON). Upon intravesical exposure, UPEC interacts with the urothelium and induces secretion of stromal cell-derived factor 1 (SDF-1/CXCL12), initiating recruitment of immune cells to the bladder [17]. This process is dependent, at least in part, on the FimH adhesin located at the tip of type 1 fimbriae [17]. In wild-type UPEC infection, bacterial hemolysinA counteracts immune accumulation by killing NK and T cells. In contrast, formaldehyde-inactivated, hemolysin-deficient UPEC allows sustained accumulation of immune cells within the bladder [15]. Bladder cancer cells exhibit elevated surface mannosylation, which enhances preferential binding of Fim ON UPEC via FimH–mannose interactions, resulting in tumor-specific bacterial attachment. This localized interaction triggers a robust immune response characterized by T cell-dependent antitumor activity, leading to eradication of non-muscle-invasive bladder tumors in preclinical mouse and rat models. NK cells may contribute inflammatory cytokines (e.g., TNF-α) following UPEC sensing; however, tumor control mediated by inactivated Fim ON is predominantly T cell-dependent. Created with BioRender.com.

To summarize the key experimental findings, we generated a schematic illustrating the proposed sequence of events underlying the antitumor activity of formaldehyde-inactivated Fim ON UPEC following intravesical administration (Figure 8). The model integrates our observations of preferential tumor binding mediated by type 1 fimbriae, local immune cell recruitment to the bladder, and the requirement for T cells in tumor eradication. The schematic highlights the distinction between wild-type UPEC and hemolysin-deficient, inactivated UPEC, and emphasizes the localized, tumor-restricted immune activation observed in the preclinical models.

## 3. Discussion

UPEC-induced urinary tract infections (UTIs) disproportionately affect women, with an incidence approximately three times higher than in men. In contrast, bladder cancer is three times more prevalent in men than in women. Given our previous findings demonstrating that UPEC can recruit immune cells to the bladder [15], we investigated whether UPEC could serve as an effective immunotherapeutic alternative to BCG for the treatment of bladder cancer.

While BCG is the current standard of care for non-muscle-invasive bladder cancer (NMIBC), it has significant limitations including limiting efficacy and substantial toxicity [8]. BCG is effective in only ~50% of patients and is associated with high rates of tumor recurrence and progression (up to 40%). It also induces adverse effects in a large proportion of patients, including dysuria, hematuria, and systemic flu-like symptoms [22]. In rare cases, BCG can cause life-threatening systemic infections [22]. Furthermore, BCG production is costly and technically complex, making it less accessible in many healthcare settings [23].

In this study, we demonstrate that intravesical treatment with UPEC, particularly when formaldehyde-inactivated, is not only safer but also more effective than BCG in preclinical bladder cancer models. Remarkably, inactivated UPEC was sufficient to cure a substantial proportion of mice bearing advanced tumors. UPEC is far easier and less expensive to culture and prepare than BCG, suggesting that this therapy could provide a more scalable and cost-effective alternative for clinical use.

Importantly, inactivated UPEC administration in mice was not associated with any observable side effects, in contrast to live-attenuated BCG, which induced toxicity including weight loss (Figure 7C, Supplemental Figure S1). As a step toward clinical translation, we quantified the residual formaldehyde levels in our preparations using a validated commercial assay. The concentration was found to be <5 ppm (<0.0005%), which is at least 40-fold lower than the levels present in many licensed human vaccines [24]. According to US FDA risk assessments, these levels are considered safe, even for infants.

A key feature of our model is that both BCG and UPEC were tested for the treatment of established tumors, rather than prophylactic settings. This allowed us to evaluate immune-mediated control in a more clinically relevant context. In immunodeficient SCID-Beige mice lacking T, B, and NK cells, neither BCG nor UPEC treatment was effective, confirming that immune cell engagement is essential for therapeutic efficacy. Further depletion experiments demonstrated that T cells—but not NK cells—are critical for mediating the antitumor effects of both therapies.

We also investigated the bacterial factors contributing to UPEC’s efficacy. Our results indicate that the type I fimbriae is partially responsible for the therapeutic effect. FIM ON UPEC, with locked expression of type I fimbriae, was more effective than the FIM OFF mutant and comparable to wild-type strains. Interestingly, although BCG also induced overall T and NK cell infiltration [12,13], it was less effective therapeutically. This discrepancy suggests that not only the magnitude, but the localization of immune cell recruitment may determine therapeutic success.

Indeed, we showed that UPEC—particularly FIM ON—bind preferentially to bladder tumor tissue in both mouse and human models via a mannose-dependent mechanism [14]. This tumor-specific targeting by UPEC may focus the immune response at the site of pathology, enhancing antitumor efficacy. Even FIM OFF UPEC retained antitumor activity and therapeutic activity comparable to BCG, suggesting that additional, yet unidentified bacterial factors mediate tumor activity.

Importantly, when UPEC was administered subcutaneously rather than intravesically, not only was therapeutic efficacy lost, but tumor progression accelerated. This likely reflects misdirected immune activation, with immune cells being recruited to the site of injection rather than the tumor itself.

Although additional functional characterization of the tumor microenvironment, such as detailed analysis of CD8^+^ and CD4^+^ T cell subsets, cytokine production, tumor histopathology, additional cytotoxic measurements, and limited mechanistic depth, could further refine our understanding of the immune mechanisms involved, these experiments could not be performed at this stage. This study was completed approximately eight years ago, and the project has since progressed to a Phase 1/2 single-arm clinical trial in patients with BCG-unresponsive non-muscle-invasive urothelial carcinoma (HOPEC; Institutional Helsinki approval no. 0459-24). Immune-profiling efforts are now being directed toward patient-derived samples within this clinical trial, which will provide mechanistic insights that are directly relevant to the therapeutic setting.

In summary, we describe a novel, effective, and safe strategy for bladder cancer immunotherapy using inactivated UPEC, a common uropathogen. This approach might offer multiple advantages over BCG, including enhanced tumor specificity, reduced toxicity, easier manufacturing, and potentially broader accessibility.

## 4. Materials and Methods

### 4.1. UPEC Inactivation

To inactivate UPEC using formaldehyde, fresh colonies of the tested UPEC (hemolysing for CFT073 and non-hemolysing for C93) were picked from blood agar plates, inoculated in LB medium (supplemented with kanamycine for C93), and grown overnight. The bacteria were pelleted by centrifugation at 4 °C and 6000 rpm for 5 min and washed twice with 30 mL of PBS. The pellet was resuspended in 10 mL 4% formaldehyde and the bacteria were incubated overnight at 4 °C for inactivation. The bacteria were pelleted by centrifugation at 4 °C and 6000 rpm for 5 min, washed three times with 30 mL of PBS sedimented, and brought to OD 600 nm = 1 in PBS.

To test killing of the bacteria by the formaldehyde treatment, 100 µL of the OD = 1 suspension were plated on LB agar plates in 1:1, 1:10, and 1:100 dilutions and incubated overnight at 37 °C.

Experiments were performed using 6- to 8-week-old C57BL/6 or Scid-Beige female mice. We used the orthotopic MB49-luc model which is transplanted into the bladder.

To ensure proper implantation of the tumors we used electro coagulation in which the mice received an electric pulse to enable tumor acceptance/implantation, especially in the bladder, where urine is present. Briefly, after a short isofluran inhalation anesthesia for induction, the mice received an i.p. injection of ketamin/xylasin for general anesthesia with addition of buprenorphine for analgesia. After shaving areas of ∼1 cm^2^ on the backs of the mice, we inserted a 24-gauge Teflon i.v. catheter (Insyte-W; Becton Dickinson, Heidelberg, Germany) transurethrally into the bladder using a lubricant (Instilla Gel; Farco-Pharma, Köln, Germany). Mice were placed with their backs on the ground plate of the cautery unit. To optimize contact, we used electrocardiogram electrode contact gel. The soft-tipped end of a spring-wire guide of a 24-gauge central venous catheter (Arrow, Erding, Germany) was inserted into the bladder via the Teflon catheter and gently pushed forward until it reached the bladder wall. The guide wire was attached to the cautery unit (Elektrotom 500; Gebrüder Martin, Tuttlingen, Germany), and a monopolar coagulation was applied for 5 s at the lowest setting (5 W). After removal of the guide wire, 0.05 mL of the tumor cell suspension was instilled. Unlike the conventional procedure, in which catheters are removed after instillation, the catheters were pinched off with a clamp, kept locked with a Luer-Lock closing cone, and left in place until the mice awakened. Using this method, we ensured a dwell time of ∼3 h. The dosing strategy for inactivated UPEC was designed to be comparable to clinically relevant BCG concentrations. In both C57BL/6 and SCID/beige mouse experiments, we used a dose of 1 × 10^8^ inactivated CFU-equivalent UPEC (bacteria), which falls within the range of BCG doses recommended by the manufacturer (2–8 × 10^8^). In the rat model, several doses of inactivated UPEC were evaluated to determine the optimal therapeutic concentration (Supplemental Figure S1).

### 4.2. Immune Cell Depletion

To deplete NK cells, mice were injected i.p with 25 μg anti-NK1.1 Ab (PK136) in 200 μL of sterile PBS. The mAb was injected 1 day prior to bacterial inoculation and then every 3 days. Similar experiments were performed in order to deplete T cells; however, for T cell depletion, an anti-CD3 antibody was used.

### 4.3. Rat Orthotopic Bladder Tumor Model (AY-27)

The efficacy and safety of inactivated UPEC were evaluated using the AY-27 orthotopic bladder carcinoma model in immunocompetent female Fischer 344 rats. Rats (9–12 per group) were anesthetized and inoculated intravesically with 1 × 10^6^ AY-27 bladder carcinoma cells. Six days post-inoculation, tumor establishment was confirmed, and animals were randomized into three treatment groups: vehicle control (PBS), Bacillus Calmette–Guérin (BCG) (1–4 × 10^7^ CFU per dose), and formaldehyde-inactivated UPEC (strain CFT073; 1 × 10^8^ CFU-equivalent per dose)

Treatments were administered intravesically twice weekly via catheterization under anesthesia for a total of five instillations. Animals were monitored daily for survival and signs of morbidity. The time to event (death or humane endpoint) was recorded, and Kaplan–Meier survival curves were plotted for each treatment group.

### 4.4. UPEC Binding Assay to AY-27 Cells

To assess UPEC binding to AY-27 cells in vitro, cells were incubated with either FIM ON or FIM OFF UPEC strains at multiplicities of infection (MOIs) of 25:1, 50:1, or 75:1 for 1 h at 37 °C. After incubation, cells were washed, fixed, and analyzed by flow cytometry to quantify bacterial attachment. Binding was expressed as mean fluorescence intensity (MFI).

### 4.5. Toxicity Assessment via Body Weight Monitoring

Body weight was measured every 3–4 days throughout the study period. Weight changes were calculated relative to baseline (day 0) to monitor systemic toxicity associated with each treatment. Data are expressed as mean ± SEM.

### 4.6. Animal Care and Monitoring

All bladder tumor induction experiments were conducted in accordance with institutional and national animal welfare regulations and were approved by the relevant ethics committee. Procedures were designed to minimize pain, suffering, and distress.

All interventions were performed under general anesthesia, with isoflurane (2–3% induction, 0.25–2% maintenance) using a non-rebreathing system, and ketamine/xylazine administered as required. Buprenorphine (0.05–0.1 mg/kg SC, twice daily) was provided for analgesia in accordance with veterinary guidelines.

Animals were monitored for general condition, behavior, posture, activity, food and water intake, respiratory status, urinary abnormalities, and body weight. Monitoring was conducted daily for the first 3 days post-procedure and at least every other day thereafter, 7 days per week, including regular body weight measurements. Animals were palpated to assess for urinary retention or urethral obstruction.

Humane endpoints were predefined and included severe or persistent distress, respiratory compromise, hematuria or blood in the cage, abdominal distension, inability to eat or drink, suspected urinary obstruction, ≥10% body weight loss between measurements or ≥20% of baseline body weight, or any other signs of pain or suffering. Animals meeting endpoint criteria were euthanized immediately.

At study completion or upon reaching humane endpoints, animals were euthanized under deep anesthesia (isoflurane or ketamine/xylazine) followed by cervical dislocation, in accordance with institutional and AVMA guidelines.

### 4.7. Statistical Methods

Kaplan–Meier survival curves with log-rank testing, or two-sided chi-square test was used as indicated in the figure legends.

## Data Availability

The data supporting the findings of this study are available from the corresponding authors upon reasonable request.

## Supplementary Materials

The following supporting information can be downloaded at: https://www.mdpi.com/article/10.3390/cells15030229/s1, Figure S1: Weight change in mice following different bacterial treatments presented in Figure 4.

## References

[1] Coley W.B. (1910). The Treatment of Inoperable Sarcoma by Bacterial Toxins (the Mixed Toxins of the Streptococcus erysipelas and the *Bacillus prodigiosus*). Proc. R. Soc. Med..

[2] Kleef R., Jonas W.B., Knogler W., Stenzinger W. (2001). Fever, cancer incidence and spontaneous remissions. Neuroimmunomodulation.

[3] Holmgren I. (1936). Employment of B. C. G. especially in Intravenous Injection. Acta Medica Scand..

[4] Babjuk M., Böhle A., Burger M., Capoun O., Cohen D., Compérat E.M., Hernández V., Kaasinen E., Palou J., Rouprêt M. (2011). EAU guidelines on non-muscle-invasive urothelial carcinoma of the bladder, the 2011 update. Eur. Urol..

[5] Siegel R.L., Giaquinto A.N., Jemal A. (2024). Cancer statistics, 2024. CA Cancer J. Clin..

[6] Ferlay J., Colombet M., Soerjomataram I., Dyba T., Randi G., Bettio M., Gavin A., Visser O., Bray F. (2018). Cancer incidence and mortality patterns in Europe: Estimates for 40 countries and 25 major cancers in 2018. Eur. J. Cancer.

[7] Zhou Y., Zhou Z., Zheng L., Gong Z., Li Y., Jin Y., Huang Y., Chi M. (2023). Urinary Tract Infections Caused by Uropathogenic *Escherichia coli*: Mechanisms of Infection and Treatment Options. Int. J. Mol. Sci..

[8] Lenis A.T., Lec P.M., Chamie K., Mshs M.D. (2020). Bladder Cancer: A Review. JAMA.

[9] Sylvester R.J., van der Meijden A.P., Witjes J.A., Kurth K. (2005). Bacillus calmette-guerin versus chemotherapy for the intravesical treatment of patients with carcinoma in situ of the bladder: A meta-analysis of the published results of randomized clinical trials. J. Urol..

[10] Larsen E.S., Joensen U.N., Poulsen A.M., Goletti D., Johansen I.S. (2020). Bacillus Calmette-Guerin immunotherapy for bladder cancer: A review of immunological aspects, clinical effects and BCG infections. APMIS.

[11] Yutkin V., Pode D., Pikarsky E., Mandelboim O. (2007). The expression level of ligands for natural killer cell receptors predicts response to bacillus Calmette-Guerin therapy: A pilot study. J. Urol..

[12] Takeuchi A., Dejima T., Yamada H., Shibata K., Nakamura R., Eto M., Nakatani T., Naito S., Yoshikai Y. (2011). IL-17 production by gammadelta T cells is important for the antitumor effect of *Mycobacterium bovis* bacillus Calmette-Guerin treatment against bladder cancer. Eur. J. Immunol..

[13] Sonoda T., Sugimura K., Ikemoto S., Kawashima H., Nakatani T. (2007). Significance of target cell infection and natural killer cells in the anti-tumor effects of bacillus Calmette-Guerin in murine bladder cancer. Oncol. Rep..

[14] Maalouf N., Gur C., Yutkin V., Scaiewicz V., Mandelboim O., Bachrach G. (2022). High mannose level in bladder cancer enhances type 1 fimbria-mediated attachment of uropathogenic *E. coli*. Front. Cell. Infect. Microbiol..

[15] Gur C., Coppenhagen-Glazer S., Rosenberg S., Yamin R., Enk J., Glasner A., Bar-On Y., Fleissig O., Naor R., Abed J. (2013). Natural killer cell-mediated host defense against uropathogenic *E. coli* is counteracted by bacterial hemolysinA-dependent killing of NK cells. Cell Host Microbe.

[16] Isaacson B., Baron M., Yamin R., Bachrach G., Levi-Schaffer F., Granot Z., Mandelboim O. (2021). The inhibitory receptor CD300a is essential for neutrophil-mediated clearance of urinary tract infection in mice. Eur. J. Immunol..

[17] Isaacson B., Hadad T., Glasner A., Gur C., Granot Z., Bachrach G., Mandelboim O. (2017). Stromal Cell-Derived Factor 1 Mediates Immune Cell Attraction upon Urinary Tract Infection. Cell Rep..

[18] Gunther N.W., Snyder J.A., Lockatell V., Blomfield I., Johnson D.E., Mobley H.L. (2002). Assessment of virulence of uropathogenic *Escherichia coli* type 1 fimbrial mutants in which the invertible element is phase-locked on or off. Infect. Immun..

[19] Snyder J.A., Haugen B.J., Lockatell C.V., Maroncle N., Hagan E.C., Johnson D.E., Welch R.A., Mobley H.L.T. (2005). Coordinate expression of fimbriae in uropathogenic *Escherichia coli*. Infect. Immun..

[20] Gunther N.W., Lockatell V., Johnson D.E., Mobley H.L. (2001). In vivo dynamics of type 1 fimbria regulation in uropathogenic *Escherichia coli* during experimental urinary tract infection. Infect. Immun..

[21] Old D.C., Duguid J.P. (1970). Selective outgrowth of fimbriate bacteria in static liquid medium. J. Bacteriol..

[22] Miyazaki J., Hinotsu S., Ishizuka N., Naito S., Ozono S., Akaza H., Nishiyama H. (2013). Adverse reactions related to treatment compliance during BCG maintenance therapy for non-muscle-invasive bladder cancer. Jpn. J. Clin. Oncol..

[23] Hatherill M., Tait D., McShane H. (2016). Clinical Testing of Tuberculosis Vaccine Candidates. Tuberc. Tuber. Bacillus.

[24] Zali S., Es-Haghi A., Ranjbar Rafie H. (2023). Validation of Free Formaldehyde Determination Method in DT Vaccine and Tetanus Toxoid Antigen. Arch. Razi Inst..

