# Supplementary material for: Targeting Bladder Cancer with Inactivated Uropathogenic E. coli: A Novel Alternative to BCG Immunotherapy
Source: Cells. 2026 Jan 26;15(3):229. doi: 10.3390/cells15030229 (PMC12897334; doi:10.3390/cells15030229)
Supplement: Supplementary file 1 [file cells-15-00229-s001.zip › cells-4092503-supplementary.pdf]

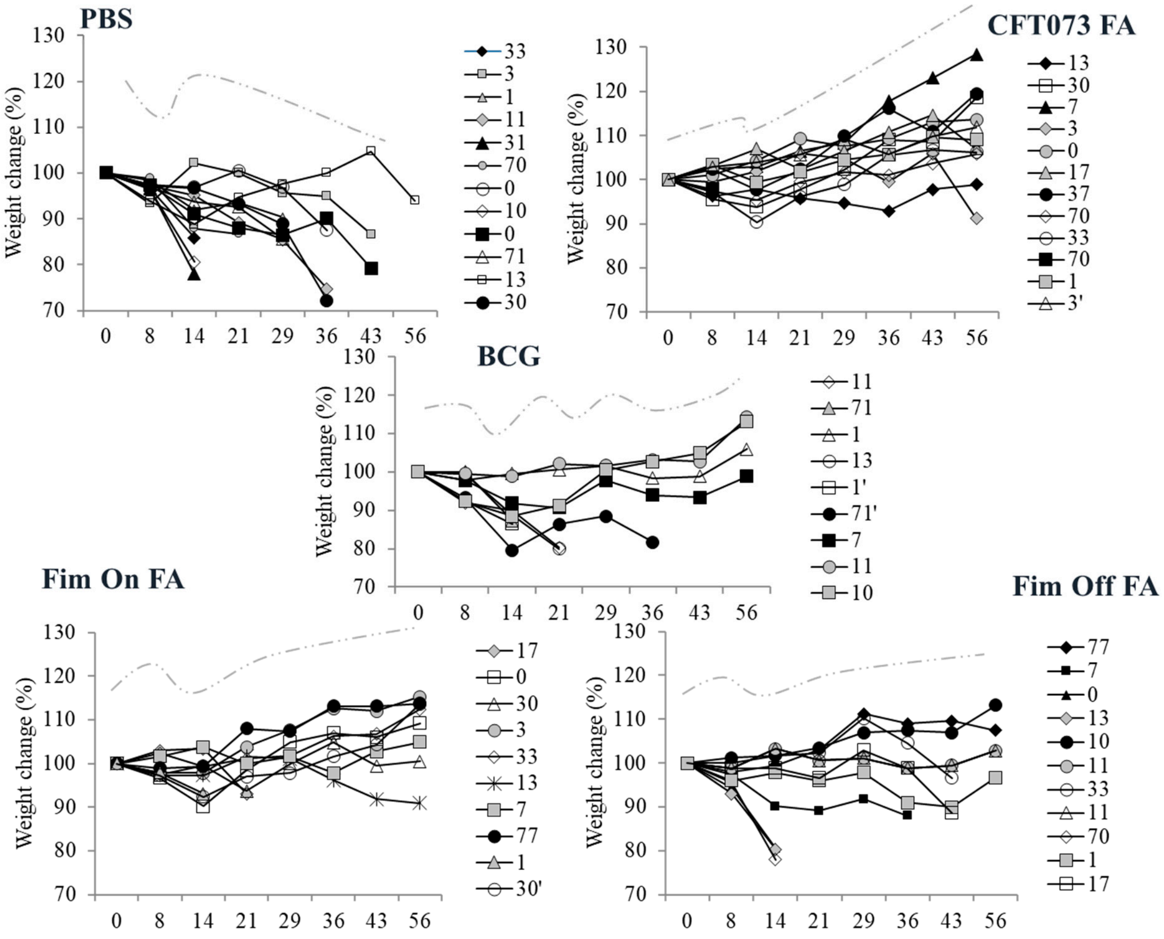

Supplemental Figure S1. Weight change in mice following different bacterial treatments presented in figure 4

Mice were treated with PBS (control), CFT073FA, BCG, Fim On FA, or Fim Off FA, and monitored for weight changes over time. Individual animals are represented by separate lines, with their ID numbers indicated. Weight change is expressed as a percentage relative to baseline (day 0). PBS and BCG groups displayed variable degrees of weight loss, with BCG-treated mice showing more pronounced toxicity. In contrast, mice treated with UPEC-derived strains (CFT073FA, Fim On FA, and Fim Off FA) maintained more stable body weights, indicating reduced systemic toxicity compared with BCG.
